# Myocarditis Following Recent Chikungunya and Dengue Virus Coinfection: A Case Report

**DOI:** 10.5935/abc.20190187

**Published:** 2019-10

**Authors:** Luís Arthur Brasil Gadelha Farias, Francisca Lillyan Christyan Nunes Beserra, Lucas Fernandes, Anderson Alesxander Rodrigues Teixeira, Juliana Mandato Ferragut, Evelyne Santana Girão, Roberto da Justa Pires Neto

**Affiliations:** 1 Universidade Federal do Ceará (UFC) - Faculdade de Medicina, Fortaleza, CE - Brazil; 2 Hospital São Carlos - Terapia Intensiva, Fortaleza, CE - Brazil; 3 Hospital São José de Doenças Infecciosas, Fortaleza, CE - Brazil; 4 Faculdade de Medicina da Universidade Federal do Ceará - Departamento de Saúde Comunitária, Fortaleza, CE - Brazil

**Keywords:** Myocarditis, Coinfection, Dengue, Chikungunya Virus

## Introduction

Dengue virus (DENV) and chikungunya virus (CHIKV) are arboviruses that cause ongoing epidemics in several countries of Latin America. DENV belongs to the *Flaviviridae* family and CHIKV is an alphavirus of the *Togaviridae* family. Both viruses are transmitted by mosquitoes of the genus *Aedes* (mainly *Aedes aegypti* and *Aedes albopictus*) and during the acute phase they may cause similar nonspecific febrile syndromes that can progress to severe or debilitating conditions.^1,2^

DENV infection has been endemic in Brazil since the 1980's. However, CHIKV is an emergent agent. Autochthonous transmission was first detected in September 2014 in the city of Oiapoque, Amapá state. Since then, there have been thousands of autochthonous cases in the country.^3^ A total of 38,499 and 277,882 cases of suspected CHIKV infection were reported by the national surveillance system in 2015 and 2016, respectively. In 2017, a total of 185,369 suspected cases were reported until December 9. Ceará (1,271 cases/100,000 inhabitants) and Roraima (789 cases/100,000 inhabitants) have the highest incidence among the Brazilian Federation states.^4^ Coinfections with these two viruses have been reported and the overall effect on the heart is still unknown.^5-7^ There have been some reports of myopericarditis following DENV and CHIKV infection, but this manifestation in coinfected patients is rare and few data are available.^8-10^

The aim of this report is to present a case of a young and immunocompetent man with myocarditis, following a recent DENV and CHIKV coinfection. We discuss the clinical course and laboratory abnormalities of this rare condition, along with its successful management in an infectious disease specialized center, highlighting the importance of being aware of this condition in developing countries endemic for DENV and CHIKV.

## Case report

The patient was a 28-year-old male, resident in Fortaleza, Ceará State and was admitted in the emergency room of São José Hospital of Infectious Diseases in May 2017. The symptoms had started 5 days prior to admission with fever, adynamia, myalgia, and worsening of general condition. He denied retroorbital pain, bleeding phenomena or abdominal pain. He had taken no medications in the previous year and had not traveled outside of Brazil. There was no previous history of cardiopathies.

Upon the initial examination, the Glasgow Coma Scale (GCS) score of the patient was E4 V5 M6. He had a heart rate of 120 beats per minute and had hypotension (70/40 mm of Hg). There was no heart murmur or pericardial rub and his pulmonary examination was unremarkable. The skin showed no rashes, and there were no petechiae or jaundice.

**Figure 1:**
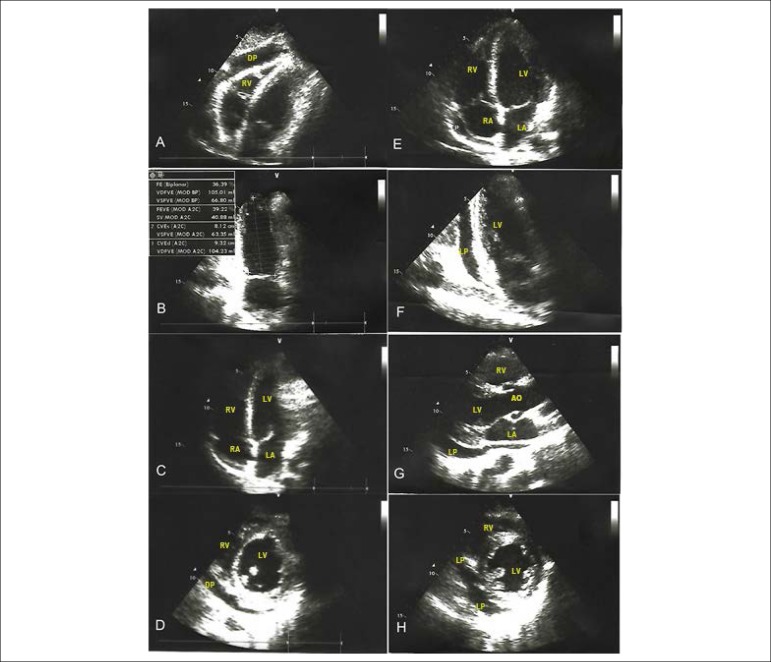
Transthoracic echocardiogram with altered left ventricular ejection fraction (36.39%), left ventricular hypercontractility, and mild bilateral pericardial effusion (A-D). E-H shows a second transthoracic echocardiogram with ejection fraction of 70%. DP and LP: pericardial effusion. RV: right ventricle; LV: left ventricle; RA: right atrium; LA: left atrium; EF: ejection fraction.

The electrocardiogram (EKG) showed supraventricular tachycardia (230 bpm) not responsive to intravenous adenosine. An electric cardioversion was performed and successfully restored the normal heart rhythm. A new EKG was performed showing ST elevation with upper concavity and PR segment depression in DII, DIII and aVF, such as ST depression in V1 and aVR. The patient was then admitted to the intensive care unit. His laboratory parameters are described in Table 1. A first transthoracic echocardiogram revealed an altered left ventricular ejection fraction (EF) (43%), left ventricular hypercontractility, and mild bilateral pericardial and pleural effusions (Figure 1). After 5 days, a new echocardiogram was performed, revealing a 36.4% EF, as well as diffuse hypokinesia and moderate pericardial effusion.

**Table 1:** Laboratory results during hospital stay of a patient with myocarditis and coinfection with dengue virus and Chikungunya virus

Characteristic	Day 1	Day 2	Day 3	Day 4	Day 5	Day 6	Day 7	Day 8	Day 9	Day 10	Day 11
Hemoglobin, g/dL	18.6	17.2	15.7	15.4	14.4	13.6	12.6	12.9	12.7	14.1	13.9
Hematocrit, %											
WBC count, x10^3^/mm^3^	12.5	17.4	19.6	18	14	11.1	9.4	8.1	6	6.1	6.7
Polymorphs, %											
Lymphocytes, %											
Lymphocyte count, x10^9^/L	1.5	1.57	1.76	1.26	1.4	1.78	1.22	1.37	1.45	1.83	2.29
Platelet count, x10^9^/l	135	122	158	177	182	169	160	156	164	199	207
Creatinine level, mg/dL	1.51	1.65	1.52	1.69	1.42	1.32	1.23	1.29	1.04	1.02	1.23
CRP levels, mg/dl	0.89	0.99	0.45	0.35	0.25	0.43	0.53	0.52	0.31	0.21	0.14
Serum Lactate level mmol/l	11.4	4.5		2.4							
NT-proBNP, pg/mL	32692	20858	14543	13832							
LDH, IU/L	522	1371		360							
ESR, mm	3			2							
CK level, U/L	1306		347								
Troponin I, ng/mL	1.05	0.5	0.47								
INR	1.56	1.73		1.51			1.26	1.19	1.14		1.12

WBC: White Blood Cell; CRP: C - Reactive Protein; NT-proBNP: N-Terminal Pro-Brain Natriuretic Peptide; LDH: Lactate Dehydrogenase; ESR: Erythrocyte Sedimentation Rate; CK level: Creatine Kinase level; INR: International Normalized Ratio.

Dobutamine (2.9 mcg/Kg/min) IV was initiated and maintained for 4 days and a single dose of 400 mg of hydrocortisone IV was administered. Treatments with immunoglobulin IV or colchicine were not considered. Paired blood cultures were negative for pyogenic agents. Antibiotics were not used.

Serologies for coxsackie virus, rubella, Chagas disease, human immunodeficiency virus, cytomegalovirus, Epstein-Barr virus (EBV), toxoplasmosis, hepatitis B virus and hepatitis C virus were negative. The patient’s serum samples were tested and found to be ELISA-IgM positive and ELISA-IgG positive for DENV and ELISA-IgM positive for CHIKV. The DENV NS1 antigen test yielded a negative result. There was a good response to therapy and the patient progressed with gradual improvement until recovery of ventricular function. After 11 days, a last echocardiogram showed an EF of 70% and persistence of pericardial effusion. The patient was discharged from the hospital 11 days after admission. Table 1 shows laboratory results during hospital stay.

## Discussion

Typical clinical manifestations of acute CHIKV infection are fever, headache, polyarthralgia/polyarthritis, myalgia, rash, and fatigue.^11^ Atypical manifestations have also been described, affecting the cardiovascular, nervous, ocular, cutaneous, and other systems.^6,12-14^ The clinical spectrum of chikungunya heart disease ranges from asymptomatic ECG alterations to potentially lethal cardiac complications.^6^

The manifestations of acute DENV infection are very similar to those of CHIKV, with a lower prevalence for joint manifestations.^15^ Heart involvement in DENV infection is not uncommon. Cardiac manifestations can vary widely, from silent disease to severe myocarditis resulting in death.^7^ Arora et al.^7^ studying 120 patients with DENV found 37.5% of patients with cardiac manifestation in the form of myocarditis and 5% with rhythm disturbance, with AV block being the most common.^16^

The specific viral diagnosis is commonly made through ELISA, a specific serological test.^11,15^ In cases of DENV, secretion of the NS1 non-structural viral protein from infected cells makes an early diagnosis possible. NS1 protein can be detected in blood and tissue samples within 9 days of the onset of fever.^15^

There is a low probability that the patient had an isolated viral infection since both viruses are part of different viral families, thus considerably reducing the cross-reaction probability. Kam et al.^17^ found that 6% of DENV-infected patients had antibodies that were cross-reactive to CHIKV.^17^ Although they share the same vector, CHIKV is part of the *Togaviridae* family, while DENV is part of the *Flaviviridae* family.

Heart disease associated with arboviruses has no specific treatment and may be a self-limited condition. Thus, quick supportive therapy to prevent further cardiac function loss and cardiogenic shock is still the most recommended management.^6^ There is also evidence that IV hydrocortisone may be helpful to accomplish full recovery in DENV myocarditis^18^ but there is yet no consensus about whether this drug should be used in this setting or if it has a real impact on recovery and mortality rates, even more in cases of combined arbovirus infection. Although arbovirus myocarditis is an acute condition, most patients persist chronically with cardiac disease, such as chronic heart failure and ECG T-wave changes.^16,19^ The role of coinfection in the severity of arbovirus cardiac manifestations is not currently known, but studies regarding other symptoms showed that it might contribute to a more severe disease.^6,7,20^ It is also noteworthy that the herein described myocarditis may have been caused solely by the CHIKV, since the NS1 protein test yielded a negative result. It is also important to note that the DENV IgM may be positive from 139 up to 179 days, respectively for secondary and primary infections.^21^

The present study has limitations. Polymerase chain reaction (PCR) was not available for the etiological diagnosis. The degree of myocardial impairment was not assessed by Magnetic Resonance Imaging (MRI). Although reported by other authors,^8,9^ MRI was not available at our center.

## Conclusion

The case presented herein suggests that DENV and CHIKV coinfection may result in myocarditis, which can be severe and may be possibly reverted with supportive therapy and correct management of cardiac function. Nevertheless, the correct etiopathogenesis of the cardiac disorder is undefined and the disease may be caused solely by either the DENV or CHIKV virus. It is important to be aware of this possible complication of arboviruses mainly in endemic areas.
